# Gasdermin as a Molecular Signature and Predictor of Adult-Type Diffuse Glioma Severity and Grading

**DOI:** 10.3390/jcm15072706

**Published:** 2026-04-02

**Authors:** Szymon Kaczor, Klepacki Hubert, Sandra Papuga, Dariusz Pawlak, Babu Harish, Adam Hermanowicz, Małgorzata Kowalska, Justyna Magdalena Hermanowicz

**Affiliations:** 1 Neurosurgery Department, Military Hospital, Kosciuszki 30, 19-300 Elk, Poland; 2 Department of Pharmacodynamics, Medical University of Bialystok, Mickiewicza 2C, 15-222 Bialystok, Polandjustyna.hermanowicz@umb.edu.pl (J.M.H.); 3 Department of Neurosurgery, Norton College of Medicine, Upstate Medical University, 603 Jacobsen Hall, 175 Elizabeth Blackwell Street, Syracuse, NY 13210, USA; 4 Department of Pediatric Surgery and Urology, Medical University of Bialystok, Waszyngtona 17, 15-274 Bialystok, Poland

**Keywords:** gasdermin, cancer, pyroptosis, biomarker, glioblastoma

## Abstract

**Background/Objectives**: Gasdermin D (GSDMD) is a critical mediator of pyroptosis—an inflammatory form of programmed cell death increasingly implicated in tumor biology. Our objective was to evaluate the utility of GSDMD as a diagnostic and prognostic biomarker and to investigate its association with tumor burden and hematological parameters. **Methods**: We analyzed GSDMD expression levels in patients with adult-type diffuse gliomas compared to healthy controls and assessed correlations with tumor size, histological grade, hematological markers, and survival outcomes. Data was complemented by transcriptomic analysis from The Cancer Genome Atlas (TCGA). Diagnostic performance was assessed using ROC curve analysis. **Results**: GSDMD expression was significantly elevated in adult-type diffuse glioma patients and increased with tumor grade, suggesting an association with disease severity. A positive correlation was observed between GSDMD level and tumor size (R = 0.332; *p* = 0.01). ROC analysis showed moderate classification ability (AUC = 0.657) with high specificity (96%), supporting its diagnostic potential. Survival analysis showed that higher GSDMD expression was associated with reduced disease-specific survival. GSDMD also correlated positively with the erythrocyte parameter mean corpuscular hemoglobin (MCH, R = 0.34, *p* = 0.016) and negatively with the systemic inflammatory marker C-reactive protein (CRP, R = −0.32; *p* = 0.042). TCGA data showed no significant sex-related differences in GSDMD expression. Baseline characteristics such as age, BMI, and coagulation parameters were matched between patients and controls. **Conclusions**: GSDMD is significantly associated with astrocytoma severity, tumor size, and inflammatory status, with elevated expression indicating a worse prognosis. Its correlation with tumor grade, survival and high specificity in distinguishing patients from healthy individuals, underlines its promise as a clinically relevant, non-sex-specific biomarker for diagnosis and monitoring.

## 1. Introduction

Biomarkers are essential tools in oncology, providing critical insights into cancer detection, progression, and therapeutic response. Adult-type diffuse gliomas remain aggressive brain tumors with limited treatment options and poor prognosis [1]. According to the 2021 WHO Classification of Tumors of the Central Nervous System, adult-type diffuse gliomas consist of three types: Astrocytoma, IDH-mutant; Oligodendroglioma, IDH-mutant and 1p/19q-codeleted; and Glioblastoma, IDH-wildtype. CNS tumor grading reflects overall expected clinical–biological behavior and is used within tumor type. It is based on histological and molecular parameters. Astrocytoma IDH-mutant tumors are graded as CNS WHO grade 2, 3, or 4, Oligodendroglioma, IDH-mutant and 1p/19q-codeleted as CNS WHO grade 2 or 3 and Glioblastoma, IDH-wildtype is graded as CNS WHO grade 4.

Despite advancements in molecular diagnostics, including the identification of IDH mutations [2] and MGMT promoter methylation [3], treatment outcomes remain suboptimal. The search for novel biomarkers with both diagnostic and therapeutic relevance is crucial for improving glioma management [4]. One emerging group of biomarkers, the gasdermin (GSDM) family, has gained attention due to its role in pyroptosis—a highly inflammatory form of programmed cell death. Unlike traditional adult-type diffuse glioma biomarkers, which primarily serve classification or predictive functions, GSDMs have the potential to influence the tumor microenvironment actively, thereby playing a dual role in both cancer characterization and treatment [5,6]. GSDMs are one of the potential biomarkers in the cell death gene category. The other known molecules are related to cell cycle or energy metabolism signal transduction [7].

GSDMD is one of the most studied members of the gasdermin family, primarily due to its ability to mediate pyroptosis and regulate immune responses. It is activated through cleavage by caspases-1, -4, -5, and -11, leading to the formation of membrane pores that result in cell lysis and the release of pro-inflammatory cytokines such as IL-1β and IL-18 [8,9,10]. This process can have profound implications for adult-type diffuse gliomas, as inflammation plays a complex role in tumor progression. While chronic inflammation often supports tumor growth, controlled activation of pyroptosis could enhance anti-tumor immunity by disrupting the immunosuppressive microenvironment characteristic of adult-type diffuse gliomas [11,12]. Furthermore, GSDMD’s involvement in neutrophil extracellular traps (NETs) suggests additional roles in immune modulation and tumor-associated inflammation, warranting further investigation into its impact on adult-type diffuse glioma progression [13,14].

Beyond GSDMD, other gasdermins, including GSDMA, GSDMB, GSDMC, GSDME, and PJVK, contribute to inflammation, cell death, and physiological functions [12]. GSDMA, primarily expressed in epithelial tissues, has been implicated in tumor suppression through its ability to induce pyroptosis [15,16]. GSDMB, which exhibits multiple isoforms with distinct functions, has been associated with both pro-tumorigenic and tumor-suppressive activities depending on the cellular context [17,18].

GSDMC, originally identified as MLZE, is unique in its nuclear and cytoplasmic localization. It is activated by caspase-8, linking it to apoptotic and necrotic pathways in addition to pyroptosis [19]. GSDME, activated by caspase-3 or granzyme B, serves as a switch between apoptosis and pyroptosis [20,21,22].

Collectively, the gasdermin family represents a promising avenue for adult-type diffuse glioma research. Their ability to induce pyroptosis and modulate inflammation distinguishes them from traditional biomarkers, positioning them as potential therapeutic targets [23]. GSDMD stands out due to its direct influence on immune responses and tumor cell death. Understanding the intricate balance between pro-tumorigenic and anti-tumorigenic inflammation will be crucial in determining the therapeutic potential of GSDMs [24].

However, further research is needed to expand our understanding of gasdermin levels in glioma patients from a clinical perspective. It is essential to investigate whether gasdermin distribution varies based on factors such as sex, body mass index (BMI), and tumor size. Additionally, examining the correlation between gasdermin concentration and tumor volume could provide valuable insights into its role in adult-type diffuse glioma progression. If successfully integrated into clinical practice, gasdermins could revolutionize glioma treatment by providing both prognostic insights and novel therapeutic strategies, ultimately improving patient outcomes.

## 2. Materials and Methods

### 2.1. Patient Cohort

In our study, we collected blood samples from 62 patients diagnosed with adult-type diffuse gliomas, confirmed by histopathological examination. The control group consisted of 26 patients admitted to the department for elective clipping of unruptured brain aneurysms without any malignant disease. All patients underwent surgery at the Department of Neurosurgery, Military Clinical Hospital in Lublin—Branch in Ełk, between March 2023 and September 2024. Tissue samples were examined and classified according to standards published in The 2021 WHO Classification of Tumors of the Central Nervous System [25].

All MRI examinations were performed on a 3.0 Tesla MRI Omega scanner from United Imaging.

We measured the tumor volume in each patient using volumetric analysis available in the Medtronic Stealth S8 planning station software (Medtronic, Minneapolis, MN 55432-5604 USA). This was performed based on images obtained from a 3 Tesla MRI scanner using a T1 3D sequence with contrast, with a 1 mm slice thickness. Then, in the Medtronic Stealthstation S8 navigation station software (Medtronic, Minneapolis, MN 55432-5604 USA), each image showing the contrast-enhanced tumor or T2 signal hyperintensities was analyzed, and the boundaries were delineated. After processing of all the tumor images, the software calculated the tumor volume.

All patients with adult-type diffuse grade 3 and 4 gliomas received Gross Total Resection of all contrast-enhancing parts of the tumor with following Stupp-Protocol Radiotherapy plus concomitant and adjuvant Temozolomide [26].

The patients included in the study did not receive corticosteroids. This eliminates a potential confounding factor and ensures that the observed levels of inflammatory markers and GSDMD reflect the underlying disease process rather than medication-induced modulation of inflammation.

The patients and the control group were analyzed for gender, age, weight, height and BMI. Blood samples were examined for PT [s], INR, APTT [s], CREA [mg/dL], CRP [mg/L], WBC [103/µL], RBC [103/µL], HBG [g/dL], HCT [%], MCV [fL], MCHC [g/dL], MCH [pg], MPV [fL], PLT [103/µL].

Preoperative blood samples were taken in a standard manner and tested in our hospital’s laboratory—Cobas pure, Roche Diagnostics, Vienna, Austria, (biochemical analysis); BC 6200, Mindray Blood, Shenzhen, China (hematological analysis).

Samples for gasdermin were collected preoperatively, placed in dedicated collection tubes, and frozen at −80 °C within 15 min.

Body mass index was calculated and obesity was classified according to commonly known standards: Class I (BMIs between 30 and 35), Class II (BMIs between 35 and 40), and Class III (BMIs above 40).

All participants provided written informed consent prior to their inclusion in the study. The research protocol was approved by the Ethics Committee of the Medical University of Białystok (approval number APK.002.172.2024), and the study was conducted in accordance with the Declaration of Helsinki.

### 2.2. Determination of the GSDM Protein Family Concentration

Serum samples were aliquoted and stored at −80 °C until analysis. No additional stabilizing buffers were added during storage. Serum GSDMA, B, C, D, and E concentrations were measured with an enzyme-linked immunosorbent assay (ELISA) according to the manufacturer’s instructions. GSDMA was provided by the Biorbyt Human Gasdermin-A ELISA kit (#orb1670316), (Biorbyt, Durham, NC 27713, USA), GSDMB by the Abbexa Human Gasdermin-B ELISA kit (abx387684), (Abbexa Ltd., Cambridge, CB4 0GJ, UK) and GSDMD by the FineTest Human GSDMD ELISA kit (EH8956), (Fine Biotech Co., Ltd., Wuhan, China). GSDMC and GSDME were purchased from the MyBiosource Human Gasdermin-C ELISA kit (MBS2881633), (MyBioSource, San Diego, CA, USA) and the Human Gasdermin-E ELISA test (MBS1607709), (MyBioSource, San Diego, CA, USA), respectively. Serum samples did not require a dilution and were estimated in a single measurement. The microplates provided in these kits were pre-coated with an antibody specific to the appropriate GSDM. Standards and samples were pipetted into the wells and any appropriate GSDM present in the sample was binded to antibodies coated on the wells. After incubation, unbound conjugates were removed by wash buffer. Then, a biotinylated detection antibody was added to bind with the appropriate GSDM conjugated on the coated antibody. After washing off unbound conjugates, Horseradish Peroxidase (HRP)-Streptavidin was added. Then, a TMB substrate was added to each well. Wells that contained target antigens, biotin-conjugated antibodies and enzyme-conjugated Avidin exhibited a change in color. The enzyme–substrate reaction was terminated by the addition of a sulphuric acid solution (stop solution). The intensity was measured spectrophotometrically at a wavelength of 450 nm using a Multiscan FC microplate reader (ThermoScientific^®^,Waltham, MA, USA). The concentration of GSDMs in the samples was determined by comparing the O.D. of the samples to the standard curve created in the SkanIt software (ThermoScientific^®^, Waltham, MA, USA). The level of GSDMs were measured in ng/mL. The amount of bound HRP or TMB conjugate was directly proportional to the concentration of GDSMA, B, C, D, and E in the sample. The standard curve ranges for GSDMA, GSDM, GSDMC, GSDMD, and GSDME were: 0.31–20 ng/mL, 0.312–20 ng/mL, 0.625–40 ng/mL, 0.156–10 ng/mL, and 0.05–15 ng/mL, respectively. The minimum detectable doses of GSDMA, GSDM, GSDMC, GSDMD, and GSDME were less than 0.1 ng/mL, 0.14 ng/mL, 0.319 ng/mL, 0.094 ng/mL, and 0.016 ng/mL. The intra-assay coefficients of variation (CV) for GSDMA, GSDM, GSDMC, GSDMD, and GSDME were CV < 8%, CV < 6%, CV < 6.4%, CV < 6.24%, and CV < 8%, respectively. The inter-assay coefficients of variation of GSDMA, GSDM, GSDMC, GSDMD, and GSDME were CV < 12%, CV < 9%, CV < 11.1%, CV < 5.91%, and CV < 10%, respectively. All laboratory measurements were conducted by a single trained operator under standardized laboratory conditions.

### 2.3. Statistical Analysis

All statistical analyses were performed using GraphPad 10 Prism (GraphPad Software, La Jolla, CA, USA). Shapiro–Wilk’s test of normality was used for data distribution analysis. The normally distributed data were analyzed using a one-way analysis of variance (ANOVA) and shown as mean ± SEM. The non-Gaussian data were presented as medians (full range) and analyzed using the nonparametric Kruskal–Wallis test. Student’s *t*-test or a nonparametric Mann–Whitney U test were used to compare differences between the glioma group and the control group. Cohen’s d was calculated for all between-group comparisons. The correlations were calculated using Spearman’s rank correlation analysis. A two-tailed *p*-value < 0.05 was considered statistically significant. A receiver operating characteristic (ROC) curve was prepared to evaluate the diagnostic performance of predictive power of the GSDMs for glioma.

We analyzed GSDMD expression levels in adult-type diffuse glioma patients compared to healthy controls and assessed correlations with tumor size, histological grade, hematological markers, and survival outcomes.

Data was complemented by transcriptomic analysis from The Cancer Genome Atlas (TCGA). The transcriptomic data were obtained from The Cancer Genome Atlas (TCGA) glioblastoma cohort (TCGA-GBM) using the cBioPortal platform (https://www.cbioportal.org). The dataset includes mRNA expression profiles derived from RNA sequencing (Illumina HiSeq; RSEM batch-normalized values, log2 value + 1) obtained from primary tumor samples collected at initial diagnosis. Clinical annotations available in the dataset included patient sex and disease-specific survival time (months). The number of analyzed samples varied depending on the availability of clinical annotations in the TCGA dataset. All analyses were performed using the datasets available through cBioPortal at the time of access. Diagnostic performance was assessed using ROC curve analysis.

## 3. Results

The cohort included individuals diagnosed with the following CNS tumors: Glioblastoma IDH-WT grade 4 (13 patients), and Astrocytoma IDH-mutant grade 3 (49 patients). All tumors were located in a single cerebral hemisphere, with no FLAIR signal abnormalities on the contralateral side. Glioblastomas (IDH-WT, grade 4) were located in the frontal lobe in four cases and in the temporal lobe in nine cases.

Astrocytomas (IDH-mutant, grade 3) were located in the frontal lobe (34 cases), temporal lobe (six cases), parietal lobe (six cases) and occipital lobe (three cases).

In all cases of Glioblastoma IDH-WT grade 4, tumor infiltration of the ependymal lining of the cerebral ventricles was not observed.

The study group included individuals aged 21 to 85 years, with a mean age of 64 years. Among them, 33 were female and 29 were male. The control group consisted of 26 patients who underwent surgery for unruptured brain aneurysms. This group consisted of individuals aged 27 to 87 years, comprising 10 females and 16 males.

Baseline characteristics of patients and controls are presented in Table 1. There was no statistically significant difference between patients and controls in terms of age and BMI, ensuring proper matching and comparability.

Statistical analysis revealed several significant differences in hematological parameters. Glioma patients (grade 3 and 4) demonstrated notably higher levels of CRP (*p* < 0.001), indicating an enhanced systemic inflammatory response within the disease group. In contrast, significantly lower values of HGB and HCT (all *p* < 0.01) were observed among patients. Coagulation parameters, including PT, INR, and APTT, showed no significant differences between the two groups, indicating that coagulation function remained largely unaffected. The results are presented in Table 1.

### 3.1. Serum Concentration of the Gasdermin Family in Glioma Patients

**Figure 1:**
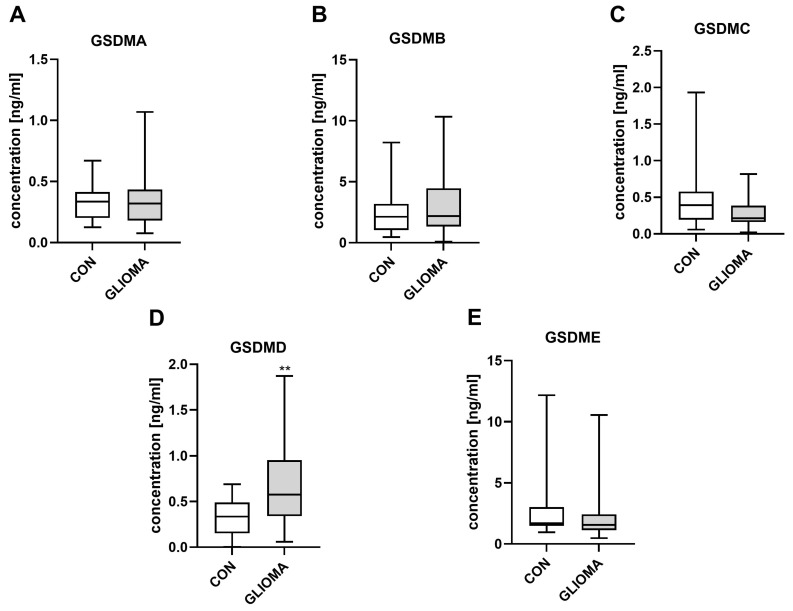
Serum levels of gasdermin (**A**–**E**) in controls (CON) and patients with glioma (GLIOMA). Data are presented as median (minimum–maximum) (CON, *n* = 26; GLIOMA, *n* = 59–62). ** *p* < 0.05 controls versus patients with glioma by Mann–Whitney U tests. Abbreviations: CON—controls, GSDM—gasdermin.

The values for GSDMA, GSDMB, GSDMC, and GSDME demonstrate no significant differences between the groups, as illustrated by the box plots (Figure 1). The analysis of the box plots indicates that the values for these proteins in both groups are similar and the ranges of variation of the results overlap, suggesting no significant differences between the study populations. The mean protein concentrations and the distribution of results do not demonstrate a clear upward or downward trend in the glioma patient group compared to the control group. However, a statistically significant elevation in GSDMD concentrations was observed in the glioma group of 0.57 (0.06–1.87), compared to the control group, with a value of 0.33 (0.004–0.69), measured with Cohen’s d = 0.18, 95% CI 0.005 to 0.34.

### 3.2. GSDMD Concentrations Based on Glioma Grade, Sex, and BMI

**Figure 2:**
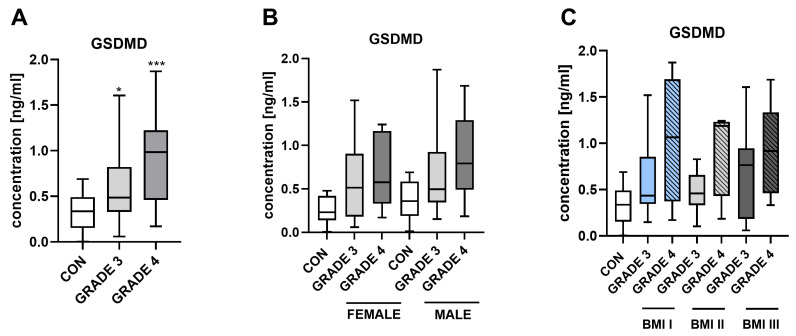
Serum levels of GSDMD in controls (CON) and patients with glioma (GLIOMA) depending on glioma grade (**A**), sex (**B**), and BMI (**C**). Data are presented as median (minimum–maximum) (CON, *n* = 26; GLIOMA, *n* = 59). * *p* < 0.05, *** *p* < 0.001 controls versus patients with glioma by one-way ANOVA test. Abbreviations: CON—controls, GSDMD—gasdermin D, BMI—body mass index.

As demonstrated in Figure 2, patients diagnosed with grade 3 and 4 gliomas exhibit higher levels compared to the control group, with statistical significance (Figure 2A). Figure 2B illustrates the relationship between GSDMD level and gender, showing that males and females with glioma have higher concentrations of this protein than the control group. Figure 2C shows GSDMD levels in patient groups classified according to body mass index (BMI I, BMI II, BMI III). The findings reveal that GSDMD levels do not attain statistical significance between BMI group and sex.

### 3.3. Analysis of the Predictive Ability of GSDMD

**Figure 3:**
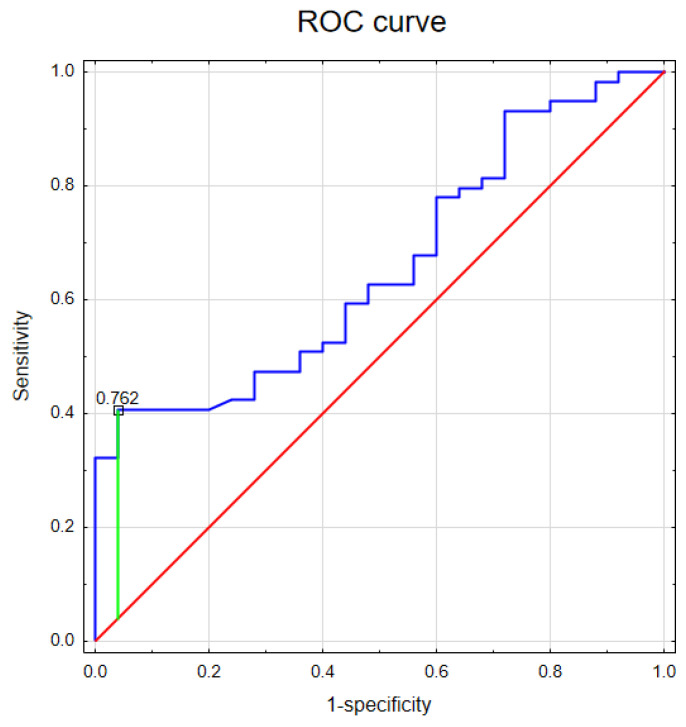
The ROC curve of the diagnostic analysis for the gasdermin D biomarker shows its potential for distinguishing adult-type diffuse glioma patients from healthy controls, *n* = 62.

ROC analysis was performed to evaluate the predictive power of the GSDMs for glioma. As shown in Table 2 and Figure 3, only GSDMD achieved statistical significance in predicting glioma and had the largest value of AUC—0.657 (95% CI 0.537–0.778), *p* < 0.01), a sensitivity of 40.7%, and a specificity of 96%.

### 3.4. Relationship Between GSDMD Concentration and Tumor Size

**Figure 4:**
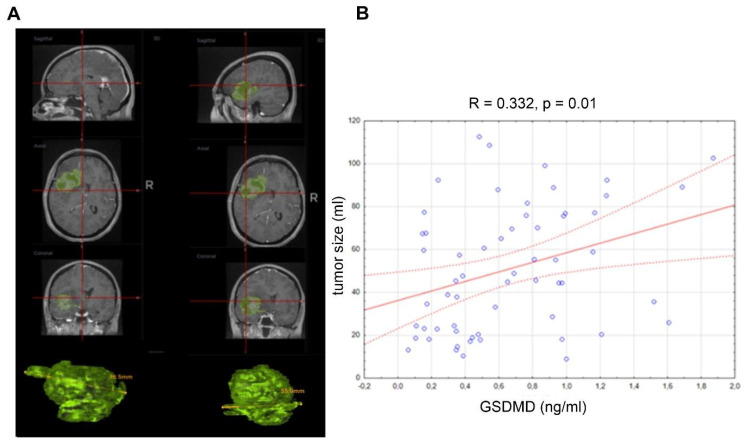
(**A**) Representative images of glioma and (**B**) correlation between serum gasdermin D (GSDMD) level and tumor size in all glioma patients (*n* = 62). For the correlation analysis between variables, Spearman’s rank-order correlation method was used.

Analysis of the association between serum GSDMD and tumor size (Figure 4) revealed a positive correlation (R = 0.332, *p* = 0.01). The Pearson correlation coefficient indicates a positive correlation between serum GSDMD levels and tumor volume, suggesting that higher GSDMD concentrations may be associated with larger tumors.

### 3.5. Correlations Between GSDMD and Laboratory Parameters

**Figure 5:**
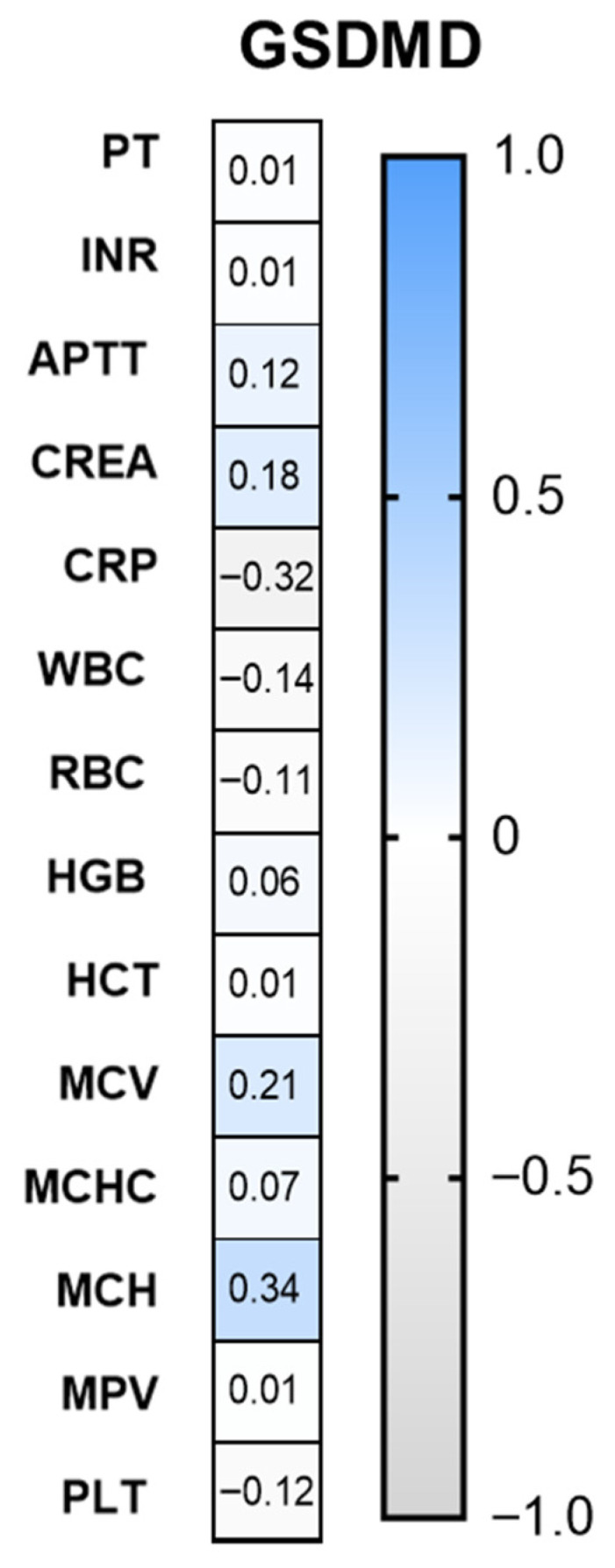
The correlation between serum gasdermin D (GSDMD) level and basic laboratory parameters in all glioma patients (*n* = 59). For the correlation analysis between variables, Spearman’s rank-order correlation method was used. Abbreviations: PT—prothrombin time; INR—international normalized ratio; APTT—activated partial thromboplastin time; CREA—creatinine; CRP—C-reactive protein; WBCs—white blood cells; RBCs—red blood cells; HGB—hemoglobin; HCT—hematocrit; MCV—mean corpuscular volume; MCHC—mean corpuscular hemoglobin concentration; MCH—mean corpuscular hemoglobin; MPV—mean platelet volume; PLT—platelet; GSDMD—gasdermin D.

Positive values, indicated by blue color in Figure 5, demonstrate a direct correlation between an increase in GSDMD expression and an increase in laboratory parameters. Conversely, negative values, represented by the gray color in Figure 5, imply a reciprocal relationship, indicating that elevated GSDMD expression is associated with a decrease in the parameter under consideration. The closer the correlation value is to 1 or −1, the stronger the relationship between the variables. Conversely, values that are proximate to zero indicate an absence of a significant correlation. A significant positive association was observed between MCH (R = 0.34, *p* = 0.016), whereas an inverse relationship was observed between GSDMD and CRP (R = −0.316, *p* = 0.042).

### 3.6. Analysis of the TCGA Database

An analysis of the Cancer Genome Atlas (TCGA) database was conducted using cBioPortal to investigate the mRNA expression of GSDMD concerning gender and months of disease-specific survival [25,26]. This analysis was based on mRNA expression levels measured in primary tumor samples collected at initial diagnosis. Detailed treatment information (e.g., type or extent of therapy) was not uniformly available in the TCGA dataset, although all patients underwent gross tumor resection. Patients under 65 years of age received 56 Gy of radiation to the tumor together with temozolomide (Temodal), while patients over 65 received reduced radiotherapy. Furthermore, TCGA data for glioblastoma primarily included samples from initial diagnosis and did not systematically capture samples from recurrent disease.

A Pearson correlation coefficient of −0.28 indicates a statistically significant (*p* = 1.20 × 10^−13^) negative relationship between GSDMD gene mRNA expression and months of disease-specific survival. This means that patients with higher GSDMD expression tend to have shorter survival, although the relationship is not strong. The very low *p*-value suggests that the result is not random and may have biological significance in the context of tumor progression.

A *p*-value of 0.418 indicates that there is no statistically significant difference in the mRNA expression of the GSDMD gene between men and women, as it exceeds the accepted significance threshold (*p* ≥ 0.05). This means that gender does not significantly affect the expression level of this gene in sequenced samples in the TCGA database.

## 4. Discussion

The study highlights the key role of gasdermin D (GSDMD) in disease severity, particularly in the context of gliomas, underlining its potential as a meaningful and clinically relevant biomarker (Figure 1). GSDMD plays a central role in pyroptosis, a cell death pathway that plays a vital role in shaping the tumor microenvironment. Its influence on immune activation, angiogenesis, and cellular proliferation suggests that GSDMD is not only a bystander, but an active participant in tumor biology [27]. Increased levels of GSDMD within the tumor microenvironment are indicative of a heightened inflammatory response, which could, in turn, foster tumor growth and increase tumor size [28].

Concerning the relationship between GSDMD levels and glioma grades, the results show a clear increase in GSDMD levels as the glioma grade increases. Specifically, patients with grade 3 and 4 gliomas exhibit significantly higher GSDMD levels compared to the control group, suggesting that GSDMD may be involved in glioma aggressiveness and could serve as a valuable biomarker for disease severity (Figure 2). This clear and statistically supported trend strengthens the rationale that GSDMD could serve as an effective marker for tumor severity. These findings were further validated by analysis of the TCGA database, which confirmed the stepwise increase in GSDMD levels from lower- to higher-grade gliomas [29]. Although our findings suggest potential links between serum GSDMD levels and tumor microenvironment-related processes, direct evidence from tumor tissue (e.g., IHC) is needed to confirm these effects. Furthermore, circulating GSDMD levels may be influenced not only by tumor expression, but also by blood–brain barrier disruption, as GSDMD activation in brain endothelial cells and microglia can promote protein release into the circulation [30,31].

The area under the ROC curve (AUC = 0.657) confirms the diagnostic relevance of GSDMD, demonstrating its ability to distinguish between glioma patients and healthy individuals. A particularly noteworthy finding is the high specificity of 96%, which ensures a low rate of false positives and highlights the accuracy of GSDMD in correctly identifying non-diseased cases. The sensitivity at the determined cut-off point (0.762) has a statistically significant *p*-value (*p* = 0.01), which reinforces the reliability and non-random nature of the observed associations. The findings provide substantial evidence in support of the utilization of GSDMD as a potential predictive biomarker in the context of glioma diagnostics. There are reports that GSDME may also be a potential biomarker of glioma [32,33], but our results did not confirm this.

**Figure 6:**
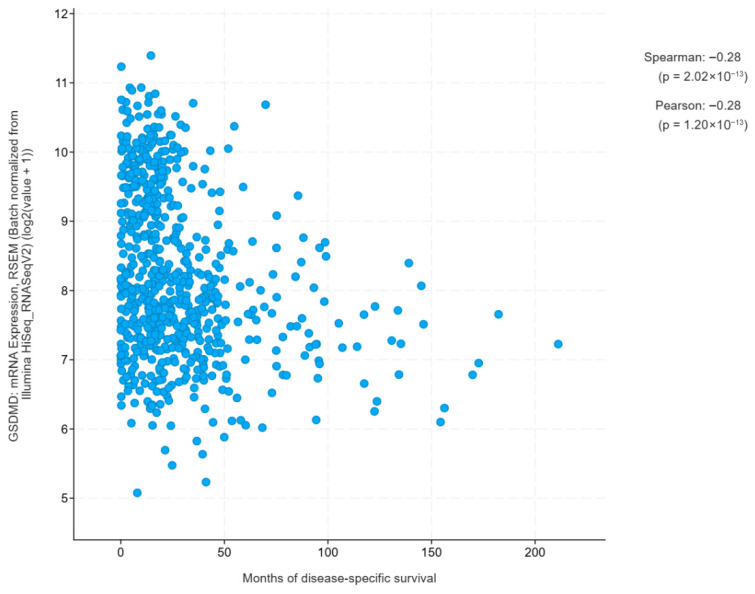
Relationship between GSDMD mRNA expression and disease-specific survival duration in the TCGA glioblastoma cohort. Each point represents an individual patient. Correlation coefficients were calculated using Pearson and Spearman tests. (*p*-value = 1.2 × 10^−13^; *n* = 671 samples).

Furthermore, analysis of disease-specific survival further confirms GSDMD’s prognostic value. Higher levels of GSDMD expression are significantly associated with reduced survival times (Figure 6), emphasizing its role not only in diagnosis but also in predicting preliminary expected survival time. Further studies are needed to investigate whether several serum levels of GSDMD analysis are reliable in monitoring disease progression and outcome.

A statistically significant positive correlation between GSDMD level and tumor size is demonstrated (R = 0.332, *p* = 0.01; Figure 4), indicating that higher GSDMD expression is linked to larger tumor volumes. This correlation serves to reinforce the rationale for considering GSDMD as a valuable biomarker for tumor management.

**Figure 7:**
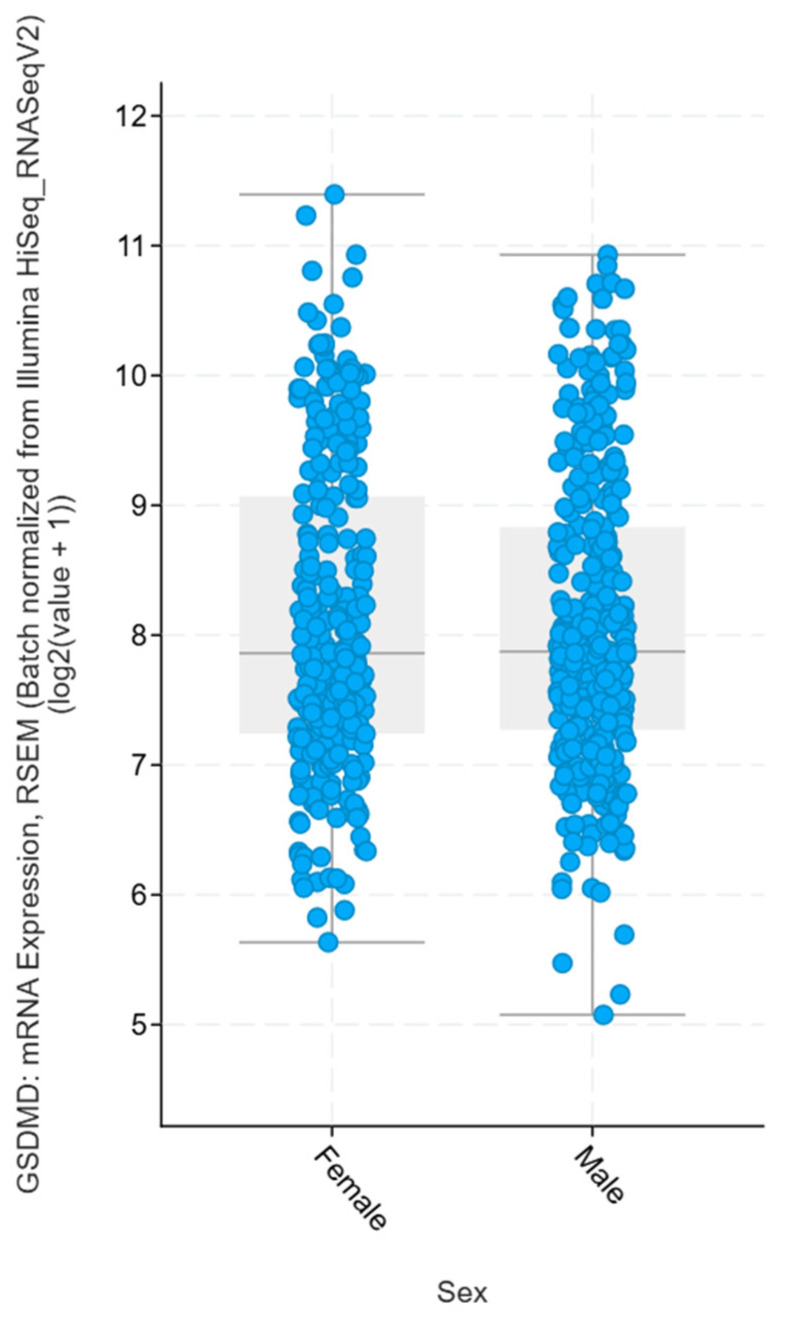
Comparison of GSDMD mRNA Expression Between Genders. (*p*-value = 0.418; *n* = 620 samples).

We have decided to analyze the TGCA database for gender differences in GSDMD expression. No statistically significant differences were observed, meaning that gender does not significantly affect the expression level of this gene, and the observed differences may be due to random variation in the data rather than actual biological mechanisms. This result suggests that GSDMD is not a sex-specific biomarker and that its role in cancers such as gliomas should be analyzed independently of the patient’s sex (Figure 7).

Regarding other biomarkers, the study finds that C-reactive protein (CRP) exhibits a negative correlation with GSDMD expression (Figure 5). This implies that as GSDMD levels increase, CRP levels, a well-known marker of systemic inflammation, may decrease. This suggests that the biomarkers may reflect distinct biological pathways that are differentially activated among patients, resulting in opposite inter-individual variability despite a shared disease-related increase, which may indicate a complex relationship between different aspects of the immune response and the inflammatory processes driven by pyroptosis. Furthermore, mean corpuscular hemoglobin (MCH) exhibits positive correlations with GSDMD levels. MCH (R = 0.34; *p* = 0.016) indicates that increased GSDMD expression may also correlate with higher hemoglobin content in individual red blood cells (Figure 4). These findings could imply a potential link between inflammation and erythropoiesis in glioma patients.

The use of aneurysm patients as controls represents a potential limitation of the present study. Future investigations including additional control groups, such as healthy volunteers or patients undergoing neurosurgery for non-inflammatory conditions, would help to further validate these findings. A further limitation is that TCGA data reflect tumor mRNA expression, while our study measured serum GSDMD protein levels, so cross-platform comparisons should be interpreted cautiously.

## 5. Conclusions

The findings reveal a consistent correlation between GSDMD and critical clinical features, including tumor grade, tumor size, and patient survival. These observations underscore the potential utility of GSDMD as a diagnostic and prognostic biomarker. Furthermore, the analysis revealed correlations between GSDMD levels and hematological parameters (CRP, MCH), indicating a complex effect of inflammation on blood morphological features. Its involvement in pyroptosis and immune modulation adds a valuable dimension to our understanding of tumor biology, and positions GSDMD as a promising target for future research and potential clinical application in the management of gliomas.

## Figures and Tables

**Table 1:** Baseline characteristics of patients and controls.

Parameter	Controls (*n* = 26)	Patients (*n* = 62)
**Sex (M/F)**	16/10	29/33
**Age [years]**	50 (27–87)	64 (21–85) NS
**BMI ratio**	25.6 (16–45)	27.6 (18–41) NS
**PT [s]**	11.26 ± 0.12	11.56 ± 0.12 NS
**INR**	1.025 ± 0.01	1.051 ± 0.01 NS
**APTT [s]**	26.54 ± 0.55	25.50 ± 0.55 NS
**CREA [mg/dL]**	0.8030 (0.5630–1.300)	0.7830 (0.4760–4.860)
**CRP [mg/L]**	0.7625 (0.50–23.80)	3.145 (0.6380–150.0) *** *p* < 0.001
**WBC [10^3^/µL]**	7.305 (4.230–17.45)	8.020 (3.250–91.76)
**RBC [10^3^/µL]**	4.732 ± 1.0	4.55 ± 0.06
**HGB [g/dL]**	14.65 ± 0.32	13.62 ± 0.2 ** *p* < 0.01
**HCT [%]**	44.83 ± 0.97	41.77 ± 0.55 ** *p* < 0.01
**MCV [fL]**	92.84 ± 0.88	91.80 ± 0.77
**MCHC [g/dL]**	32.67 ± 0.14	32.58 ± 0.08
**MCH [pg]**	30.32 ± 0.29	31.15 ± 0.66
**MPV [fL]**	10.96 ± 0.24	10.38 ± 0.19
**PLT [10^3^/µL]**	244.0 (107.0–410.0)	250.5 (119.0–541.0)

NS—non-significant; M/F—male/female; BMI—body mass index; PT—prothrombin time; INR—international normalized ratio; APTT—activated partial thromboplastin time; CREA—creatinine; CRP—C-reactive protein; WBCs—white blood cells; RBCs—red blood cells; HGB—hemoglobin; HCT—hematocrit; MCV—mean corpuscular volume; MCHC—mean corpuscular hemoglobin concentration; MCH—mean corpuscular hemoglobin; MPV—mean platelet volume; PLT—platelet. ** *p* < 0.01, *** *p* < 0.001 controls versus patients with glioma.

**Table 2:** Summary of statistical analysis values for GSDMD.

	AUC Value (95%CI)	Proposed Cut-Off	Sensitivity (%)	Specificity (%)	*p*-Value
GSDMD	0.657 (0.537–0.778)	0.762	40.7	96%	<0.01

## Data Availability

The data generated in this study are available upon request from the corresponding author.

## Abbreviations

The following abbreviations are used in this manuscript:

ANOVA	Analysis of Variance
APTT	Activated Partial Thromboplastin Time
AUC	Area Under The Curve
BMI	Body Mass Index
CNS	Central Nervous System
CON	Controls
CRP	C-Reactive Protein
ELISA	Enzyme-Linked Immunosorbent Assay
FLAIR	Fluid-Attenuated Inversion Recovery
FMF	Familial Mediterranean Fever
GBM	Glioblastoma
GSDM	Gasdermin
GSDMA	Gasdermin A
GSDMB	Gasdermin B
GSDMC	Gasdermin C
GSDMD	Gasdermin D
GSDME	Gasdermin E
HGB	Hemoglobin
HCT	Hematocrit
HRP	Horseradish Peroxidase
IDH	Isocitrate Dehydrogenase
INR	International Normalized Ratio
MCH	Mean Corpuscular Hemoglobin
MCHC	Mean Corpuscular Hemoglobin Concentration
MCV	Mean Corpuscular Volume
MGMT	O6-Methylguanine-DNA Methyltransferase
MPV	Mean Platelet Volume
MRI	Magnetic Resonance Imaging
NETs	Neutrophil Extracellular Traps
PLTs	Platelets
PT	Prothrombin Time
RBCs	Red Blood Cells
ROC	Receiver Operating Characteristic
SEM	Standard Error of the Mean
TCGA	The Cancer Genome Atlas
TMB	Tetramethylbenzidine
WBCs	White Blood Cells
WHO	World Health Organization
